# Creating an Interprofessional guideline to support patients receiving oral anticoagulation therapy: a Delphi exercise

**DOI:** 10.1007/s11096-019-00844-0

**Published:** 2019-05-15

**Authors:** Nikki L. Damen, Bart J. F. Van den Bemt, Kurt E. Hersberger, John Papastergiou, Filipa Alves da Costa, Silas Rydant, Naomi S. Wartenberg, Trudy Lobban, Isabelle Arnet, Sotiris Antoniou, International Pharmacists for Anticoagulation Care Taskforce (iPACT)

**Affiliations:** 1 Netherlands Institute for Health Services Research (NIVEL), Utrecht, The Netherlands; 2 Sint Maartenskliniek Nijmegen, Nijmegen, The Netherlands; 3 Radboud Univerity Medical Center Nijmegen, Nijmegen, The Netherlands; 4 University of Basel, Basel, Switzerland; 5 University of Toronto, Toronto, Canada; 6 Centro de Investigação Interdisciplinar Egas Moniz (CiiEM), Instituto Universitário Egas Moniz, Caparica, Portugal; 7 Royal Pharmacist Association of Antwerp (KAVA), Antwerp, Belgium; 8 Barts Health NHS Trust, London, UK; 9 UCLPartners, London, UK

**Keywords:** Guideline development, Interprofessional guideline, Oral anticoagulations, Pharmaceutical care

## Abstract

*Background* Oral anticoagulation therapy has proven beneficial impact on the prevention of thromboembolic events. However, the use of antocoagulatns also increases the risk of bleeds. To maximize the benefits and minimize the risks of the treatment, guidance on appropriate use of oral anticoagulants is essential. An international guideline describing relevant components and requirements for pharmaceutical care for patients receiving a therapy woth oral antocoagulants would increase the quality of care. However, recommendations on pharmaceutical care for patients on anticoagulation is lacking. *Objective* This study aims to develop an interprofessional guideline to support patients in their use of oral anticoagulation therapy. *Method* Two systematic literature searches were performed on existing guidelines on the management and interventions to improve-oral anticoagulant use, to generate possible recommendations. Subsequently, an international expert panel with 26 pharmacists with extensive experience in clinical and/or scientific work on anticoagulation from a total of 22 European and 4 non-European countries was constituted. With this (geographically well distributed) expert panel, a four-round internet-based Delphi technique was conducted to reach consensus on their relevance. Items were ranked on a 1–10 scale of agreement. A median agreement score of ≥ 7.5 was considered the threshold for consensus. Levels of importance were rated on a 1–3 scale. *Setting* A global network of 26 pharmacists specialized in oral antocoagulation therapy. *Main outcome measure* Development of inter-professional guideline. *Results* After the four Delphi rounds 18 guideline recommendations were formulated. Consensus of opinion was achieved for all recommendations (median agreement: 8.5–10.0), whereas mean levels of importance were between 1.1 and 2.0 (SD: 0.2–0.7). The following domains were rated as most important targets for improving the care around oral anticoagulation: ‘INR-monitoring’, ‘Transfer of care between health care settings’, ‘Adherence to medication’, ‘Patient communication and engagement’, and ‘Medication reconciliation and medication review’. *Conclusion* The 18 recommendations included in this guideline provide the base for optimization of anticoagulation care across different countries/healthcare systems. Future work involves translating the guideline recommendations into clinical practice. Once implemented, the recommendations of the guideline will support health care providers with the pharmaceutical care for patients on, oral anticoagulation which will improve the effective and safe use of these medicines.

**Electronic supplementary material:**

The online version of this article (10.1007/s11096-019-00844-0) contains supplementary material, which is available to authorized users.

## Impacts on practice

- There are 18 recommendations in a new guideline, which provide the base for optimization of oral anticoagulation care for patients across different countries and health care systems.
- The most important elements for improving the care around anticoagulation are: ‘INR-monitoring’ ‘Transfer of care between health care settings’ ‘Adherence to medication’ ‘Patient communication and Engagement’ and ‘Medication Reconciliation and medication review’.
- The guideline recommendations must be translated into clinical practice. Multidisciplinary training, sharing best-practices and coaching involved health care providers, could help overcome implementation problems and improve success rate.

## Introduction

Oral anticoagulation therapy (OAT) is one of the most common pharmacological interventions for patients with cardiovascular diseases. Currently, about 2% of the population in developed countries receives vitamin K-antagonists (VKAs) for the prevention of thromboembolic events. Within the last decade, non-vitamin K antagonists (NOACs) have emerged as alternatives to VKAs. Overall, oral anticoagulation prescriptions are expected to increase rapidly worldwide due to the aging population [1, 2].

Although OAT has beneficial effects on the prevention of thrombotic events and improving long-term survival, the use of these medications is not without risk. Inhibiting the coagulation cascade to reduce thrombosis consequently increases the risk of serious gastrointestinal and intracranial bleeds. Most emergency hospitalizations for recognized adverse drug events in older adults result from a few commonly used medications, with a substantial proportion of these events being related to VTA-use and bleeding complications being the most common reason for medication-related hospital admissions [3–6].

Due to the serious nature of the drug-related problems (DRPs) associated with OAT, health care professionals and patients need to be actively supported to ensure safe and effective medication use. Several studies have illustrated that inappropriate prescribing, monitoring, and administration of OAT occur frequently. Oral anticoagulants are often underdosed, inadequately monitored, inappropriately stored, and not taken as prescribed, all of which contribute to the increased risk for DRPs [7–10].

Both the European Heart Rhythm Association (EHRA) and the European Society of Cardiology (ESC) included updated adequate structured follow-up of OAT-patients as essential for patient safety in the updated versions of their guidelines [11, 12]. Various health care providers are involved in providing anticoagulation care, including physicians, nurses and pharmacists. Efficient multidisciplinary collaboration and communication is therefore essential to ensure patient safety. Nonetheless, this collaboration is often suboptimal which can exaggerate uncertainties including confusion in the division of responsibilities and frequent miscommunications [13].

An international guideline describing relevant components and requirements for adequate structured follow up for patients receiving oral anticoagulation therapy would increase the quality of pharmaceutical care for patients on OATS, foster consistency in the provision of pharmaceutical care including continuity of care [11, 12, 14]. However, an interprofessional guideline to support medication use in patients using OAT is currently lacking.

## Aim of the study

This study aims to develop an interprofessional guideline to support patients in their use of Oral anticoagulation therapy (OAT).

## Ethics approval

An ethics approval for the described processes was not required. All participants of the Delphi-procedure agreed to par-ticipate in the research. The data are reported on group-level, so individual participants can not be identified from the results.

## Method

### Study design

**Fig. 1:**
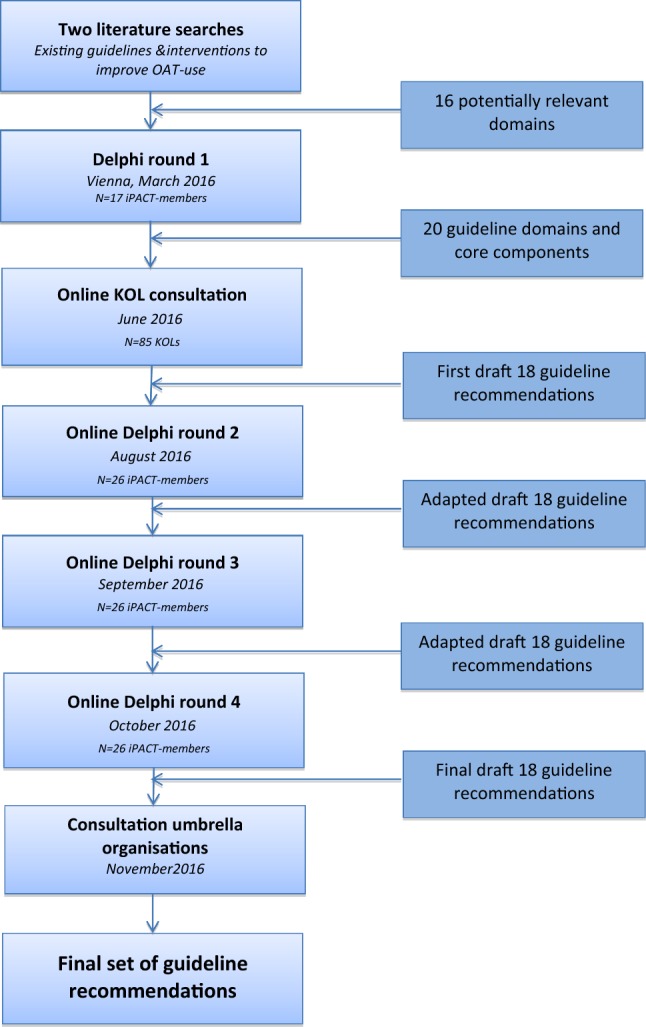
Overview of our Delphi technique

To obtain insight into existing recommendations on the management of patients receiving OAT and evidence on interventions to optimize medication use in this patient group, two systematic literature searches were performed. The first aimed at critically appraising existing guidelines, standards, and quality measures on OAT, whereas the second evaluated the impact of interventions to improve OAT-use. Combining the results of the two reviews, a list of potential domains to be reflected upon in the guideline were subjected to a consensus method. Accordingly, an internet-based Delphi exercise was conducted with international OAT-experts, to develop internationally applicable and acceptable interprofessional guideline recommendations to support patients receiving OAT (Fig. 1).

### Literature searches

#### Existing guidelines

To obtain insight into existing guidelines on the management of OAT patients, a literature search was conducted in PUBMED, EMBASE, CINAHL, the Cochrane library, and Global Health searched up until March 2015. In this literature review, OAT guidelines, standards, and quality measures published up until March 2015, and with full-text written in English, were included. The detailed search strategy can be found in “Appendix 1” section.

A manual query was performed using reference lists of included studies. In order not to exclude potential ‘grey literature’, websites of international societies and health care organizations were searched to retrieve additional OAT-related guidelines, treatment standards, and quality measures.

Full-text manuscripts, fulfilling the above inclusion criteria, were evaluated using the methodological tool, AGREE II to assess quality [15]. Only guidelines receiving a high score in the overall quality assessment (i.e., 4–7) were used for further analyses. Of the included documents, all recommendations and other information related to OAT were extracted. This information was organized into a “grid” according to the domains and standards of the Foundation Pharmacy Framework of the Royal Pharmaceutical Society [16]. The design (and results) of this literature review will be published separately.

#### Interventions to optimize OAT-use

To obtain insight into the current evidence on interventions to improve OAT-use, a second systematic literature search was conducted. PUBMED (including MEDLINE) and EMBASE were searched up until March 2016. The search strategy included terms related to pharmaceutical care interventions combined with OAT-use. The detailed search strategy can be found in “Appendix 1” section.

Studies were eligible for inclusion if they focussed on pharmaceutical care interventions for patients receiving OAT (NOACs or VKAs), and compared the effectiveness (e.g., time-in-therapeutic range (TTR)) and/or safety (e.g., bleeding, thromboembolic events) of the pharmaceutical care intervention with usual care. Two reviewers independently performed title/abstract and full-text selection, quality assessment (using the STROBE statement), as well as data extraction of the eligible studies. The design (and results) of this systematic literature review will be published separately.

Subsequently, the interventions found to improve OAT-use were linked to existing guideline recommendations as retrieved in the first literature review. Based on this combination of results, domains of interprofessional OAT-care potentially relevant for inclusion in the interprofessional guideline to support patients receiving oral anticoagulation therapy were identified.

### Delphi exercise

The Delphi exercise was initiated in March 2016 and comprised four internet-based rounds with an international expert panel as well as two broader consultation rounds of international ‘key opinion leaders (KOLs)’ and global umbrella organizations, all of which were finalised by November 2016.

The expert panel of the first Delphi exercise was comprised of the ‘International Pharmacists for Anticoagulation Care Taskforce (iPACT)’. iPACT consists of 26 pharmacists with extensive experience in clinical and/or scientific work on OAT from a total of 22 European and 4 non-European countries. In the selection procedure for the experts it was assured that all continents were represented, the European experts were geographically well distributed and experts with knowledge on specific relevant subthemes (adherence (e.g. IA, FA), monitoring (e.g. JP, SA), education (FA, IA, SR)) were represented. The project team, consisting of two independent researchers (NW and ND) and a project leader/pharmacist (BvdB). All electronic questionnaires were constructed using ‘Google forms’ and two email reminders were issued to the panel members each round.

In accordance with previous research, mean and median levels of agreement were reported and a threshold for consensus of 75% was adopted. [17, 18] A consensus was achieved if the median level of agreement of the expert panel members on a certain topic was 7.5 or higher [17, 18].

In Fig. 1, an overview of our Delphi exercise is provided. Delphi round 1 comprised a 1-day meeting with the iPACT expert panel in Vienna (Austria). In semi-structured group discussions, the domains from the literature searches were discussed and revised, and corresponding core components (e.g., how, when, and by whom should the OAT-care activity be performed) were formulated.

The results of Delphi round 1 were verified by a broader international online expert panel. For this purpose, iPACT members provided nominations for KOLs in each of their respective countries. Nominations were provided across even different anticoagulation-related disciplines: pharmacists, physicians, nurses, pharmaceutical companies, patient organizations, anticoagulation services, and general practitioners. Based on their input, the project team translated the domains and core components into a first draft of guideline recommendations on interprofessional OAT-care.

In Delphi round 2, an online questionnaire was constructed to present the first draft of the guideline recommendations to iPACT-panel members. In addition to soliciting suggestions on complementing and/or rephrasing the recommendations, experts were asked to rate their level of agreement with (the content and relevance of) each recommendation on a 10-point Likert scale (1 = completely disagree’ to 10 = completely agree’) as was done in previous studies [17, 18].

In the online questionnaire of Delphi round 3, we first provided the iPACT-expert panel members with feedback on results of Delphi round 2. This feedback was provided by means of bar graphs of panel ratings and information on the mean/median level of agreement per recommendation. iPACT-experts were then asked to rate their level of agreement with the adapted recommendations on a 10-point scale. Panel members could also provide suggestions for complementing and rephrasing the recommendations.

In the final Delphi round 4, we again provided the iPACT-panel members with feedback on the results of the previous round. In addition, they were asked to rate each of the recommendations on their level of importance using a scale of 1–3: 1=’A must have for the final set of recommendations’; 2=’Would be nice to have in the final set of recommendations’; 3=’Is not important in a final set of recommendations’ [17, 18]. Further, there was the possibility to provide final remarks on the proposed wording of the recommendations.

As a last step, in a second broader consultation round the final draft of recommendations were presented to several umbrella organizations relevant for OAT-care (i.e., American Society of Health-system Pharmacists (ASHP), Anticoagulation Europe, American College of Clinical Pharmacy (ACCP), European Association of Hospital Pharmacists (EAHP), EHRA, European Stroke Association (ESA), ESC, European Society of Clinical Pharmacy (ESCP), European Society for Patient Adherence, COMpliance, and Persistence (ESPACOMP), International Society on Thrombosis and Haemostasis, and Pharmaceutical Group of the European Union (PGEU)), as suggested by iPACT-experts. By means of a final online questionnaire we asked contact persons of these organizations to provide us with their feedback on the guideline.

## Results

### Literature searches

#### Existing guidelines

For the first literature search on existing guidelines on the management of OAT patients, 6777 titles and abstracts were screened, of which 30 full-text articles were included in the final selection of studies. The preliminary framework, to which the overview of existing guideline recommendations was translated, resulted in 12 standards subdivided in 45 dimensions for interprofessional care application in patients receiving OAT.

#### Interventions to improve OAT-use

Of the 11,171 titles and abstracts screened, 125 full-text articles were included in the final selection of studies. All relevant information on interventions to improve OAT-use was extracted. From the results of this literature review, 12 different interventions (e.g., patient education, self-management, medication review, medical training) were identified, each having a substantial impact on the effectiveness and safety of OAT as demonstrated by, for example, a greater TTR and/or fewer bleeding and thromboembolic events.

In combining the results of both literature searches, every intervention found to improve oral anticoagulant use could be linked to one or more existing guideline recommendations as found in the first literature search. Based on this combination of results, and without rejecting any guideline or intervention, 16 unique domains potentially relevant for interprofessional care in OAT-patients were eventually extracted by the project team. These unique domains provided the base for the Delphi exercise (Table 1).

**Table 1:** Input for Delphi round 1: 16 domains relevant for interprofessional OAT-care, based on the literature reviews

Domains for interprofessional oat-care		
1. Adherence-interventions	7. Treatment plan	12. Transfer of care between health care settings
2. Shared-care/self-management	8. Pharmacogenetic dosing	13. Medical training
3. Patient education	9. Pharmacotherapeutic surveillance	14. Process management
4. Anticoagulation pharmacist/nurse	10. INR-monitoring, patient self-testing, point-of-care testing	15. Pharmacy workforce
5. Telemedicine	11. Medication supply	16. Governance
6. Medication review/clinical rules		

*OAT* oral anticoagulation therapy

##### Delphi round 1

Seventeen members (65%) of the iPACT-expert panel attended the 1-day meeting in Vienna. During this meeting, the 16 domains as identified in the literature searches were critically revised in semi-structured group discussions. After both plenary and subgroup discussions, a final set of 20 domains relevant for interprofessional OAT-care was determined. Three additional domains were added to the original set based on expert opinion: ‘Lifestyle and cultural-specific aspects’, ‘Screening’, and ‘Patient communication and engagement’. Further, the domain ‘Pharmacogenetic dosing’ was divided into ‘Pharmacogenomics’ and ‘Metabolic monitoring’, resulting in the final total of 20 domains. In addition, several titles of domains were adapted (e.g., ‘Adherence-interventions’ was changed into ‘Adherence to medication’) to better reflect clinical practice of interprofessional OAT-care (Table 2).

**Table 2:** Result of Delphi round 1: 20 domains relevant for interprofessional OAT-care, as determined by the iPACT-expert panel

Domains for interprofessional oat-care		
1. Adherence to medication	8. Medication reconciliation and medication review	15. Medication supply
2. Lifestyle and cultural-specific aspects	9. Therapy plan	16. Transfer of care between health care settings
3. Patient communication and engagement	10. INR-monitoring, patient self-testing, point-of-care testing	17. Governance
4. Patient education	11. Screening	18. Continuing professional development
5. Shared-care/self-management	12. Pharmacovigilance	19. Pharmacy workforce
6. Anticoagulation pharmacist/nurse	13. Pharmacogenomics	20. Process management
7. Telemedicine	14. Metabolic monitoring	

*OAT* oral anticoagulation therapy

During the second half of the meeting, panel members split into six subgroups and discussed the core components of the 20 domains (e.g., how, when, by whom should the intervention to improve OAT-use be performed). To enable additional reflection, after the in-person meeting these core components were further enhanced and clarified by email. This allowed for any non-attendees to be involved in the process. They had the opportunity to also reflect on the identified set of domains and core components.

##### Consultation of international key opinion leaders

To verify the results of Delphi round 1, we approached 85 KOLs of 14 (both European and non-European) countries. In total, 26 KOLs (30.6%) responded to our online questionnaire, and provided suggestions for complementing and/or rephrasing of our 20 domains and core components.

Based on KOLs’ feedback, the core components of the domain ‘Process management’ were integrated into the domains of ‘Governance’ and ‘Pharmacy workforce’, as these were highly overlapping. In addition, the domain ‘Metabolic monitoring’ was deleted as its core components could be integrated in the ‘Therapy plan’ domain.

##### Online Delphi round 2

Of the 26 iPACT-panel members, 20 completed the online questionnaire (response rate: 76.9%) of Delphi round 2. The mean level of agreement ranged from 7.6 to 9.2 [SD: 0.8–2.6]. The median level of agreement varied between 8.0 and 10.0 for all guideline recommendations, indicating that all recommendations met the criteria for inclusion in this second round (Appendix 2 of electronic supplementary material). Comments on complementing and rephrasing of the guideline recommendations were built into the next version.

##### Online Delphi round 3

Similarly, 20 out of 26 (76.9%) iPACT-experts completed the online questionnaire of Delphi round 3, which included feedback on the results of Delphi round 2. The mean level of agreement with the 18 adapted guideline recommendations ranged from 7.4 to 9.4 [SD: 0.7–2.0]. Experts’ comments on complementing and rephrasing were built into an adapted draft of the guideline recommendations.

##### Online Delphi round 4

In this final Delphi round, 20 out of 26 (76.9%) online questionnaires were completed. The mean level of importance—as rated by the experts on a 1–3 scale—ranged from [1.1 to 2.0], with standard deviations ranging between [0.2 and 0.7]. There was one final comment, which the project team processed into the final draft of the guideline recommendations.

##### Second consultation round of global umbrella organizations

The input of the broader consultation of different umbrella organizations relevant for OAT-care, was built into the final set of guideline recommendations.

In Table 3, the Top 5 interprofessional guideline recommendations to support patients receiving oral anticoagulation therapy are presented. Specifications of these recommendations are listed in Appendix 2 of electronic supplementary material, in which the final set of 18 guideline recommendations is presented. In Appendix 2 of electronic supplementary material, the main recommendation, specifications, corresponding mean/median level of agreement (LoA) of Delphi round 3, and level of importance (LoI) of Delphi round 4 is presented.

**Table 3:** Top 5 interprofessional guideline recommendations to support patients receiving oral anticoagulation therapy

	Main recommendation
1. INR-monitoring	In patients using VKAs, the international normalized ratio (INR) should be monitored regularly to ensure the safety and effectiveness of oral anticoagulation therapy (OAT)
2. Transfer of care between health care settings	Accurate information about patients’ OAT, including current medications, should be transferred accurately between different health care settings to ensure seamless care
3. Adherence to medication	In patients using oral anticoagulation medication, adherence to and persistence with therapy should be assessed and supported. Patients and their caregivers should be educated on adherence
4. Patient communication and engagement	Communication with and the involvement of patients and their caregivers should be considered an integral component of safe and effective interprofessional OAT-care
5. Medication reconciliation and medication review	In OAT-patients, medication reconciliation and medication review should be performed on a regular basis to ensure the safe, effective, and clinically appropriate use of medication

## Discussion

In this four-round internet-based Delphi exercise (preceded by two systematic literature searches), 18 recommendations for better and safer use of OATS were formulated. These recommendations were formulated by a multidisciplinary team across different countries/healthcare systems which reached consensus on all recommendations. The Top 5 interprofessional guideline recommendations comprised ‘INR-monitoring’, ‘Transfer of care between health care settings’, ‘Adherence to medication’, ‘Patient communication and Engagement’, and ‘Medication Reconciliation and medication review’ (details listed in Table 3 and Appendix 2 of electronic supplementary material).

Compared to the other recommendations, expert consensus on the agreement/importance ratings of ‘Pharmacogenetic assessment’ and ‘Screening’ varied greatly. This could be explained by the fact that both topics are currently subject to debate and countries differ in their opinion on whether to incorporate these interventions in standard clinical OAT-care. For each recommendation, the median consensus threshold of ≥ 7.5 was reached after Delphi round 2, so they were included in the final guideline.

In translating our guideline recommendations into frontline clinical practice, two important prerequisites should be taken into account. First, with the guideline recommendations we aim to address interprofessional, multidisciplinary OAT-care rather than care provided by any specific healthcare professional, such that recommendations are applicable to the broader field of OAT-care. Second, we acknowledge the fact that important elements of OAT-care may differ between countries and local contexts. Notwithstanding, the Delphi technique included representation from many countries and a variety of healthcare professionals and patient care organisations in order to mitigate for these differences.

### Strengths and limitations

The Delphi exercise is a well-recognized, structured process designed to achieve group-consensus on certain topics. A Delphi exercise include its ability to easily involve individual experts across different geographical and clinical settings. Further, subject anonymity is an important prerequisite of the Delphi, reducing the effects of dominant individuals on a group-based process [17, 19].

An additional advantage of our Delphi exercise is the online procedure we used. Panel engagement over the four online survey rounds was high, with response rates of > 70% and a large volume of comments and feedback being reported in every round. This advantage of online data collection have been reported previously [20].

Several limitations of the Delphi exercise should also be acknowledged. First, given the internet-based character of the Delphi, there was an inability for expert panel members to meet and discuss uncertainties or ambiguities in the construction or wording of the questionnaires. Another weakness of this consensus methodology is that the success of the Delphi process depends on the panel chosen. There are no universally agreed criteria for the selection of experts, nor agreement on the minimum or maximum number of members needed. When the iPACT-expert panel was formed, specific attention was paid to a representative distribution across countries. The final panel comprised only of pharmacists, as pharmacists are fully specialized in pharmaceutical care. One might argue that this could hamper the generalizability of the guideline to other OAT health care professionals. Therefore, we verified our results in two broader consultation rounds with KOLs from different anticoagulation-related disciplines and umbrella organizations. As these two rounds did not result in changes to the recommendations, our guideline seems to be generalizable to other disciplines as well.

## Conclusion

In the current Delphi exercise an interprofessional guideline to support patients receiving oral anticoagulation therapy was developed. The 18 recommendations included in this guideline provide the base for optimization of OAT-care for patients across different countries and health care systems. Future work involves translating the guideline recommendations into clinical practice with an assessment on the impact to patient care.

### Electronic supplementary material

Below is the link to the electronic supplementary material.

Supplementary material 1 (DOCX 36 kb)

## Appendix 1

### Search strategies

#### Search strategy for the literature review on existing guidelines

The following keywords were used: “guideline”, “clinical practice guideline”, “treatment guideline”, “guidance”, “recommendation”, “standard of care”, “quality measure”, and “oral anticoagulation” “Standards” OR “Standard”[Mesh] OR Pharmaceutical care” OR Pharmaceutical Services”[Mesh] OR “Pharmaceutical service” OR “Pharmaceutical services” OR “Quality” OR “Quality”[Mesh] OR “Measure” OR “Measure”[Mesh] OR “Quality Measure” OR “Quality Measure”[Mesh], Educational OR Education OR “Pharmacist Education as Topic”[Mesh] OR “Health Education”[Mesh] OR “Pharmacist knowledge”[Mesh] OR Knowledge OR “Anticoagulant care” OR “Anticoagulation care” OR “Anticoagulation services” OR “Anticoagulation service” OR “Patient safety” OR “Patient safety”[Mesh], “Pharmacy” OR “Pharmacy”[Mesh] OR Pharmacist[Mesh] OR Pharmacist[Text Word] OR “Pharmaceutical Services”[Mesh], “Atrial” OR “Atrial”[Mesh] OR “Fibrillation” OR “Fibrillation”[Mesh] OR “AF” OR “AF”[Mesh]

AND

Anticoagulants[Mesh] OR “Oral anticoagulants” OR “Oral anticoagulant” OR “Oral anticoagulant”[Text Word] OR “Oral anticoagulants”[Text Word] OR Phenindione OR Phenindione[Mesh] OR Phenindione[Text Word]“Vitamin K antagonist” [Mesh] OR “Vitamin K antagonist”[Text Word] OR Warfarin OR Warfarin[Mesh] OR Warfarin[Text Word] OR Acenocoumarol OR Acenocoumarol[Mesh] OR Acenocoumarol[Text Word] OR “Non vitamin K antagonist”[MESH] OR “Non vitamin K antagonist [Text Word] OR Direct Oral Anticoagulant [MESH] or Direct Oral Anticoagulant [Text word] OR Apixaban Or Apixaban [Supplementary Concept] OR Apixaban[Text Word] OR Dabigatran OR Dabigatran[Mesh] OR Dabigatran[Text Word] OR Edoxaban OR Edoxaban [Supplementary Concept] OR Edoxaban[Text Word] OR Rivaroxaban OR Rivaroxaban[Mesh] OR Rivaroxaban[Text Word]

#### Search strategy for the literature review on interventions to optimize OAT-use

Rivaroxaban OR Rivaroxaban[Mesh] OR Rivaroxaban[Text Word] OR Apixaban Or Apixaban [Supplementary Concept] OR Apixaban[Text Word] OR Edoxaban OR Edoxaban [Supplementary Concept] OR Edoxaban[Text Word] OR Acenocoumarol OR Acenocoumarol[Mesh] OR Acenocoumarol[Text Word] OR Phenprocoumon OR Phenprocoumon[Mesh] OR Phenprocoumon[Text Word] OR Warfarin OR Warfarin[Mesh] OR Warfarin[Text Word] OR Dabigatran OR Dabigatran[Mesh] OR Dabigatran[Text Word] OR Ximelagatran OR Ximelagatran [Supplementary Concept] OR Ximelagatran[Text Word] OR Anticoagulants[Mesh] OR “Oral anticoagulants” OR “Oral anticoagulant” OR “Oral anticoagulant”[Text Word] OR “Oral anticoagulants”[Text Word] OR Phenindione OR Phenindione[Mesh] OR Phenindione[Text Word]

AND

(“Observational Study” OR “Observational Study”[Publication Type] OR “Observational Studies as Topic”[Mesh] OR “Randomized Controlled Trial”[Publication Type] OR “Randomized Controlled Trial” OR “Randomised controlled trials” OR “Randomized Controlled Trials as Topic”[Mesh] OR “Controlled Clinical Trial” [Publication Type] OR “Controlled Trial” OR “Controlled trials” OR “Non-Randomized Controlled Trials as Topic”[Mesh] OR “Retrospective study” OR “Retrospective Studies”[Mesh] OR “Prospective study” OR “Prospective Studies”[Mesh] OR “Longitudinal Studies”[Mesh] OR “Longitudinal study” OR “Longitudinal studies” OR “cohort study” OR “cohort studies” OR “Cohort Studies”[Mesh])

AND

“Medication review” OR “Medication reviews” OR “Drug Utilization”[Mesh] OR “Drug Utilization Review”[Mesh] OR Pharmacogenetic* OR Pharmacogenetics[Mesh] OR Genotype OR Genetic* OR Adherence OR Medication Adherence[Mesh] OR Persistence OR Compliance OR”Patient Compliance”[Mesh] OR Pharmacist* OR Pharmacist[Mesh] OR Pharmacist[Text Word] OR “Pharmaceutical Services”[Mesh] OR “Pharmaceutical service” OR “Pharmaceutical services” OR Educational OR Education OR “Patient Education” OR “Patient Education as Topic”[Mesh] OR “Health Education”[Mesh] OR “Patient medication knowledge”[Mesh] OR Knowledge OR “Pharmaceutical care” OR “Patient care” OR “Point-of-Care Systems”[Mesh] OR “Point-of-care” OR “Anticoagulant care” OR “Anticoagulation care” OR “Anticoagulation services” OR “Anticoagulation service” OR “Drug-Related Side Effects and Adverse Reactions”[Mesh] OR “Patient counseling” OR continuation OR discontinuation OR Monitoring OR Self-management OR “medication errors” OR “medication error” OR “medication errors”[MeSH] OR “Patient safety” OR “Patient safety”[Mesh]
