# Supplementary material for: Creating an Interprofessional guideline to support patients receiving oral anticoagulation therapy: a Delphi exercise
Source: Int J Clin Pharm. 2019 May 15;41(4):1012–20. doi: 10.1007/s11096-019-00844-0 (PMC6677675; doi:10.1007/s11096-019-00844-0)
Supplement: Supplementary file 1 — Supplementary material 1 (DOCX 36 kb) [file 11096_2019_844_MOESM1_ESM.docx]

**Appendix 2 .**Result of Delphi round 4: 18 interprofessionalguideline recommendations for patients receiving oral anticoagulation therapy(Mean/median level of agreement (LoA) of Delphi round 3, and level of importance (LoI) of Delphi round 4)

| **Interprofessional guideline to support patients receiving oral anticoagulation therapy** | | | |
| --- | --- | --- | --- |
| **1. SHARED CARE/ SELF-MANAGEMENT** | **Mean LoA: 9.1±0.7** | **Median LoA: 9.0** | **Mean LoI: 1.1±0.3** |
| ***Main recommendation:***  **Patients and their caregivers should have access to appropriate and consistent information and support, to make informed choices about the use of oral anticoagulation, the implications of choosing not to take them, and the possibility of self-managing oral anticoagulation therapy (OAT).**  *Specifications:*   - In the shared-care process, patients and their caregivers (e.g., family members, helpers) should be placed at the centre of care. One of the key roles of the team of health care professionals (e.g., pharmacist, physician, nurse) is to enable them to become as autonomous as possible in their OAT- management - Patients should be provided with the option to manage their own OAT (self-testing, self-interpretation, and/or in the case of VKAs self-dosing). Patients’ competency and willingness to self-manage should be assessed prior to initiation of therapy - Patients should be provided with appropriate education materials on self-management (including, if applicable, self-monitoring and self-dosing) *(see also recommendation 3)* - In the shared-care and self-management process, the health literacy level of patients and their caregivers should be considered and they should be supported with appropriate information resources - In addition, patient preferences, clinical features, lifestyle, and cultural-specific aspects should be considered in treatment choices *(see also recommendation 5)* | | | |
| **2. PATIENT COMMUNICATION AND ENGAGEMENT** | **Mean LoA: 9.3±0.8** | **Median LoA: 9.0** | **Mean LoI: 1.1±0.3** |
| ***Main recommendation:***  **Communication with and the involvement of patients and their caregivers should be considered an integral component of safe and effective interprofessional OAT-care.**  *Specifications:*   - Pharmacists and other health care providers should be trained in effective patient communication regarding OAT - Patients should be provided with advice, information, and educational materials to aid their understanding of all aspects of OAT. This should be done for individual patients as well as specific patient groups - In addition, patients’ caregivers should be identified and provided with specificinformation regarding OAT - Communication could take place by any available means, such as face-to-face discussion and contact by telephone, text, and/or e-mail - A clear line of communication should be ensured for emergency situations, especially outside primary care providers’ operating hours - Patients and their caregivers should be actively involved in discussions upon OAT-initiation and follow-up - Health care providers should listen to patients and their caregivers to understand their preferences, concerns, and beliefs regarding OAT, and address them appropriately - In addition, lifestyle, cultural-specific, and clinical aspects should be considered in the communication with patients and their caregivers *(see also recommendation 5)* - To be able to communicate effectively about OAT with patients who do not speak the native language, patient information resources should be available in a language suitable for the patient (and/or their caregivers). This could be provided in any combination of verbal, written, and/or visual (e.g., pictograms) forms | | | |
| **3. PATIENT EDUCATION** | **Mean LoA: 9.3±0.9** | **Median LoA: 9.5** | **Mean LoI: 1.1±0.3** |
| ***Main recommendation:***  **Patients and their caregivers should be provided with - and always have access to - clear, structured, understandable, and evidence-based information on OAT to ensure safe and effective medication use. This information should be tailored to patients’ needs.**  *Specifications:*   - Patients and their caregivers should be educated on the *benefits* (e.g., prevention of atrial fibrillation (AF)-related stroke, deep venous thromboembolism, and pulmonary embolism) and potential *risks* (e.g., haemorrhages) of OAT - Patients and their caregivers should be educated on the prevention and detection of potential complications (e.g., AF-related stroke, haemorrhage, deep venous thromboembolism, and pulmonary embolism) of OAT - Patients and their caregivers should be educated on how to act in emergency situations. In this, the need for using anticoagulation alert cards and INR booklets/diaries should be emphasized - In addition, during a patient education session the following aspects should be discussed: the reason for being prescribed OAT, duration of therapy, therapy monitoring (INR), adherence to therapy, specific aspects with respect to food intake (e.g., diet, alcohol consumption, and Ramadan), drug and food-drug interactions, side effects, and specific situations leading to therapy suspension (e.g., surgery) - The fact that OAT is not a *cure* for AF but significantly reduces the risk of AF-related complications (e.g., stroke, deep venous thromboembolism, and pulmonary embolism) should be emphasized - Patients should be provided with appropriate education materials (e.g., leaflets, medication cards, video materials, and websites) to ensure safe OAT-use. VKA-patients could be specifically provided with educational material facilitating INR-monitoring (e.g., INR-score in writing available to the patient) and enabling the follow-up of dose adjustments in the case of self-management - The provided information should be in line with current guidelines and adapted to patients’ preferences and clinical features - Patients and their caregivers should receive appropriate verbal and written information, tailored to patients’ individual needs, at initiation of therapy, the first anticoagulation clinic or follow-up appointment, and throughout the course of therapy (including alert cards) - Patients and their caregivers could be pointed at authentic, professionally-verified internet sources to retrieve adequate information on OAT - Patient information resources should be available in multiple languages. Where necessary, the use of translators and /or pictograms could be considered - The level of understanding of counselling information should be assessed, for example, by asking patients and/or their caregivers to repeat or paraphrase the information provided (redundancy). The health literacy level of patients and their caregivers should be considered, and appropriate information resources should be provided. In addition, methods of assessing how much the patient understands of his/her education (e.g., post-counseling questionnaires) could be considered | | | |
| **4. INDIVIDUALIZED THERAPY PLAN** | **Mean LoA: 8.8±1.1** | **Median LoA: 9.0** | **Mean LoI: 1.3±0.4** |
| ***Main recommendation:***  **To maximize the effect and minimize the risks of OAT, an appropriate individualized therapy plan should be provided to each patient. This therapy plan should be periodically reviewed throughout the course of anticoagulation therapy.**  *Specifications:*  THERAPY PLAN:   - The therapy plan should be provided to patients by responsible health care providers, and the providers’ names and contact information should be included in the therapy plan - In the therapy plan laboratory results (e.g., haemoglobin, renal function, hepatic function, and for VKA-patients also INR results), medication history and current medication list (including over-the-counter (OTC) medication), clinical characteristics (e.g., weight, age, indication), pharmaceutical formulation, and patient preferences should be considered. These parameters should be included to ensure appropriateness of OAT and individualized dosing if indicated - The therapy plan should also address temporary cessation planning (as well as re-initiation), including a communication plan that ensures that health care providers who might amend the therapy plan communicate these changes to other health care providers responsible for the patients’OAT-care   THERAPY PLAN REVIEW:   - The patients’ therapy should be reviewed at least every six months by a responsible health care provider, balancing between risks and benefits of OAT, using appropriate stroke and bleeding risk score systems (e.g., CHA_2_DS_2_-VASc) - Modifiable bleeding risk factors (e.g., hypertension, renal disease, medication associated with bleeding, alcohol usage, stroke history, risk of falls and home hazards) should be reviewed at least every six months and addressed as appropriate - The date of the last review should be noted in the therapy plan | | | |
| **5. LIFESTYLE, CULTURAL-SPECIFIC, AND CLINICAL ASPECTS** | **Mean LoA: 9.1±0.8** | **Median LoA: 9.0** | **Mean LoI: 1.5±0.5** |
| ***Main recommendation:***  **Lifestyle, cultural-specific, and clinical aspects should be considered in the management of OAT-patients.**  *Specifications:*   - Regarding lifestyle and cultural-specific aspects, specific attention should be paid to diet, physical activity, working conditions, socioeconomic status, social support, household, and cultural beliefs and behaviours - Regarding clinical characteristics, patient disabilities (e.g., hearing, visual, and/or cognitive impairment), learning difficulties, co-medication, and co-morbidities should be considered when assessing ways to support OAT-patients to achieve better outcomes - Elderly patients, patients with caregivers, patients with short-term memory loss, patients with poor health literacy, and any language barriers such as foreign patients from abroad may need additional support to improve their OAT - Culturally appropriate counselling tools might be helpful to address differences between cultural groups - The geographical availability of anticoagulation services and patients’ capability to reach these services easily should be considered in OAT-management | | | |
| **6. ADHERENCE TO MEDICATION** | **Mean LoA: 9.4±0.8** | **Median LoA: 10.0** | **Mean LoI: 1.1±0.2** |
| ***Main recommendation:***  **In patients using oral anticoagulation medication, adherence to and persistence with therapy should be assessed and supported. Patients and their caregivers should be educated on adherence.**  *Specifications:*   - Patients’ personal motivation for, beliefs on, and experiences with taking their oral anticoagulation medication as well as practical barriers should be explored *regularly* to assess the impact on short- and long-term adherence - Medication adherence should be assessed by using effective and understandable (basic) tools, such as validated patient questionnaires (e.g., Morisky-8), refill rates, specific lab control, and/or electronic devices (including apps) - In assessing patients’ adherence, the number of medications the patient is expected to take should be considered, as polypharmacy can impact on adherence to the anticoagulationregimen - In addition, adherence assessment should consider potential difficulties in daily routine affecting adherence as well as key factors on *when* and *how* to take oral anticoagulation medication in relation to food - To support patients’ adherence, oral anticoagulation regimens should be as simple as possible (i.e., simplified dosing regimen) and appropriate aids (e.g., compliance packaging, and medication calendar) and/or graphical charts should be provided to patients - In case of decreased adherence, responsible health care providers should try to identify and understand the reason for this decrease, and focused adherence support should be provided - Patients and their caregivers should also be educated on specific situations that may lead to dose changing or temporary therapy cessation (i.e., surgery). The importanceofre-initiationOAT after a temporary cessation should be emphasized. If needed, an anticoagulation expert (e.g., pharmacist, physician, or nurse) should be consulted in these specific situations | | | |
| **7. INR-MONITORING** | **Mean LoA: 9.3±1.0** | **Median LoA: 9.5** | **Mean LoI: 1.1±0.2** |
| ***Main recommendation:***  **In patients using VKAs, the international normalized ratio (INR) should be monitored regularly to ensure the safety and effectiveness of OAT.**  *Specifications:*   - INR-monitoring should be easily accessible and performed by either point-of-care testing (POC, self-testing) or laboratory testing. Laboratory testing should be performed by health care providers experienced in OAT-management (e.g., anticoagulation services, pharmacists, and nurses) - Independent of the INR-monitoring system chosen, the system should be easily accessible, cost-effective, follow a structured protocol, and be audited frequently. In addition, self-testing devices should be assessed regularly for accuracy - When self-testing and self-dosing, patients and their caregivers should be provided with advice, education, dosing instructions, standardized software, and supervision to ensure safety and appropriate OAT-dosing - The dosing of oral anticoagulation medication should be adjusted in response to the INR-measurements. Other medications, adherence to medication, alimentation potentially affecting INR-values as well as other parameters (e.g., patients’ wellness, clinical situation, laboratory measurements, blood pressure, liver and renal function) should be considered - A standard procedure should be in place for determining time-in-therapeutic range (TTR) and follow-up measurements, with the minimum of 65% used as a validated method - TTR, frequency of high and low INR’s, frequency of dose changing, and frequency of adverse events could be considered indicators of therapy quality - Details about the INR-monitoring should be clearly documented in patient notes, anticoagulation booklets, and/or electronic records or software | | | |
| **8. PHARMACOGENETIC ASSESSMENT** | **Mean LoA: 7.7±2.0** | **Median LoA: 8.5** | **Mean LoI: 2.0±0.6** |
| ***Main recommendation:***  ***Pharmacogenetic testing may be considered in selected high-risk patients to ensure safe and effective OAT-dosing.***  *Specifications:*   - High-risk patients are, for example, patients with INR-control problems, patients vulnerable to cytochrome P (CYP) interactions, and patients with a high bleeding risk - In patients with a high bleeding risk, pharmacogenetic assessment should ideally be performed *before*OAT-initiation | | | |
| **9. TRANSFER OF CARE BETWEEN HEALTH CARE SETTINGS** | **Mean LoA: 9.4±0.9** | **Median LoA: 10.0** | **Mean LoI: 1.1±0.2** |
| ***Main recommendation:***  **Accurate information about patients’ OAT, including current medications, should be transferred accurately between different health care settings to ensure seamless care.**  *Specifications:*   - Patients and their caregivers should be advised whom to contact if they need more information or support regarding their OAT when transferred to another health care setting. This should include information on who will monitor the continuing therapy and how to access a continued supply of their anticoagulation medication - For patients moving between different health care settings, arrangements should be in place to ensure a safe supply of oral anticoagulation medication, and on-going support and monitoring if necessary - Upon referral of patients for disease management, there should be a clear documented process (supported by standard protocols) which should include information on: patient identification, contact details, primary disease, current medications, reason for OAT, duration and required target therapy range, relevant medical history, any medication allergies, co-existing diseases, responsibilities of the different health care providers, and previous anticoagulation use - Anticoagulation service providers should manage patients’ risk profiles and ensure a clear line of communication with primary health care providers on ‘concerns and actions’ (e.g., missed appointments, poor adherence, and concerns over risk/ benefit balance) - Common patient records could be helpful to record and retrieve relevant OAT-related patient information, including information on patient referrals within different health care settings. These patient records should be accessible to both patients and their different health care providers - Medication reconciliation should be conducted at every transition of care (e.g., at hospital admission and hospital discharge) to assure safe and effective OAT*(see also recommendation 10)* - Patients themselves should always have an anticoagulation card on them to inform the health care team about the risk of bleeding in case of an emergency (redundancy). In addition, patients should keep a follow-up diary, possibly electronic, in which the main information about their OAT is summarised (e.g., INR-monitoring, dose adjustments, and side effects) | | | |
| **10. MEDICATION RECONCILIATION AND MEDICATION REVIEW** | **Mean LoA: 9.4±0.9** | **Median LoA: 10.0** | **Mean LoI: 1.1±0.3** |
| ***Main recommendation:***  **In OAT-patients, medication reconciliation and medication review should be performed on a regular basis to ensure the safe, effective, and clinically appropriate use of medication.**  *Specifications:*  MEDICATION RECONCILIATION   - Medication reconciliation could be performed by any responsible health care provider (e.g., pharmacist, nurse, physician) - Medication reconciliation should be performed at least once a year and at *every* transition of care (e.g., hospital admission, transfer from the emergency department or intensive care to another hospital ward, and hospital discharge) to obtain and verify a complete and accurate list of patients’ current medications   MEDICATION REVIEW   - Medication review could be performed by pharmacists, physicians, or nurses, according to the local guidelines - Medication review should be performed in partnership with patients and their caregivers - Medication review should be performed *before*OAT-prescription and thereafter at least once a year, to ensure that no clinically significant interactions exist between patients’ OAT and other medications - It is desirable to perform an additional medication review at *every* medication change (e.g., addition of a new drug) as well as in high-risk situations such as adverse drug reactions and hospital admissions - During medication review, interactions between OAT and food/herbs, medication side-effects, and level of medication adherence should also be checked - Medication review should include all types of medication in their review including prescribed, OTC, traditional, and complementary medications (e.g., herbal products and supplements) - Medication review should also include recent POC-/laboratory measurements (e.g., blood results) and other parameters (e.g., age, gender, and bodyweight) - Any consequent changes should be the result of consultation with the patient and/or the physician, and should be recorded in the patients’ records | | | |
| **11. MEDICATION SUPPLY** | **Mean LoA: 9.5±1.0** | **Median LoA: 10.0** | **Mean LoI: 1.3±0.5** |
| ***Main recommendation:***  **OAT-patients should have a continuous supply of oral anticoagulation medication, including appropriate dosing instructions, to ensure safe and effective therapy.**  *Specifications:*   - Patients’ OAT-prescriptions should be reviewed regularly to ensure safe medication supply, and to reduce the risk of omitted and/or delayed medication administration - For a given patient, a stable supply in the selected dosage form should be maintained - Dosing information should be visually displayed in both milligrams and number of tablets, to enhance appropriate medication intake and to improve adherence - Fluctuations in INR-levels and clinically significant interactions with other medications should be considered, and dosing strengths should be adapted accordingly - In addition, medication adherence and the incidence of OAT-related adverse events should be monitored *(see also recommendation 6 & 13)* - If patients require a monitored dosage system for their regular medication, risk assessment should be conducted upon dispensing their oral anticoagulation medication via such a system. Use of a monitored dosing system for VKAs need to allow for the flexibility of dosing VKAs, required for maintenance of optimum INR. When considering NOACs, stability within any system needs to be ensured - If needed, additional support of a pharmacist or nurse should be in place to ensure the safe availability of OAT | | | |
| **12. TELEMEDICINE** | **Mean LoA: 8.2±1.7** | **Median LoA: 8.5** | **Mean LoI: 1.9±0.4** |
| ***Main recommendation:***  **OAT-patients should be offered telemedicine as a service, in order to support them in their medication use and to give them remote access to care.***  *Specifications:*   - Once OAT is successfully initiated, patients should be offered telemedicine services by health care providers specialized in OAT*(see also recommendation 17)* - Telemedicine services should include both advice on and monitoring (e.g., INR values) of patients’ OAT - Telemedicine services should also evaluate patients’ adherence to OAT*(see also recommendation 6)* - Telemedicine services should include access for patients to their own personal medical record (including INR results and dosing history) and online educational resources - Telemedicine services could be offered by telephone (including both calls and text messages), e-mail, video, or other electronic services (e.g., apps)   * According to the World Health Organisation (WHO), telemedicine can be defined as *“the delivery of health care services - where distance is a critical factor - by all health care professionals using information and communication technologies for the exchange of valid information for diagnosis, treatment, and prevention of disease and injuries, research and evaluation, and for the continuing education of health care providers, all in the interests of advancing the health of individuals and their communities”* | | | |
| **13. PHARMACOVIGILANCE** | **Mean LoA: 9.3±0.7** | **Median LoA: 9.0** | **Mean LoI: 1.2±0.4** |
| ***Main recommendation:***  **The incidence (if any), prevalence, and recurrence of OAT-related adverse events should be determined, monitored, and reported.**  *Specifications:*   - Responsible health care providers should monitor OAT-related adverse events. In case of an adverse event, they should report them to the appropriate authorities (e.g., treating physician and national pharmacovigilance centre) - Monitoring OAT-related adverse events is essential for risk minimisation strategies for newly introduced anticoagulation medication. Therefore, patients new on OAT should be closely monitored to identify clinically relevant adverse events and drug interactions - By reporting adverse events, as many details as possible should be provided, including concomitant diseases, laboratory measurements, time to onset of adverse event, OAT-duration, and patients’ evolution. This contributes to determining the level of causality between OAT and the adverse event (causal, probable, or possible) | | | |
| **14. SCREENING** | **Mean LoA: 7.4±2.3** | **Median LoA: 8.5** | **Mean LoI: 1.8±0.7** |
| ***Main recommendation:***  **Patients with risk factors (e.g., age > 65 years, diabetes, hypertension, or common AF symptoms) not receiving OAT should be routinely screened on AF, and (if needed) referred to a physician for diagnosis.**  *Specifications:*   - Screening for AF could be performed by any trained health care provider in primary care settings (e.g., by community pharmacist, and/or general practitioner) as well as by physicians in hospital settings - Screening for AF could be carried out by any appropriately validated method, including the use of electronic devices to enhance the reliability of detection (e.g., mobile app) - When AF is found to be present at screening, patients should be referred to their treating physician for diagnosis. The health care provider who performed the screening procedure should advise the patient of the risks of not completing the follow-up process | | | |
| **15. GOVERNANCE** | **Mean LoA: 9.1±1.1** | **Median LoA: 9.0** | **Mean LoI: 1.2±0.4** |
| ***Main recommendation:***  **A governance framework (e.g., clinical guidelines, audits, and standard operating procedures (SOPs)) should be developed to ensure safe and effective OAT-management. This framework should reflect current guidelines, safe practices, and patient surveys.**  *Specifications:*   - A central governance body should be in place in each country or region, responsible for ensuring consistent policies and practices, and with performance-related oversight and the power to remove privileges if needed - These central regulatory agencies should be encouraged to acknowledge the role of health care professionals (e.g., pharmacists and physicians) in anticoagulation services, *and* modify/ update their regulations accordingly - High standards of practice should be ensured for all pharmaceutical services delivering care to OAT-patients - Written (preferably multidisciplinary) procedures and clinical protocols for the delivery of optimized OAT should be established and updated periodically if necessary - Procedures for patient assessment, adjustment of OAT-dosing, supporting of adherence, and scheduling of OAT-follow-up and monitoring should be implemented - In addition, procedures for the management, documentation, and referral of unexpected events (e.g., bleeding, clotting, other potential OAT-related adverse effects, or medical problems not related to OAT) should be in place - Pharmaceutical services involved in OAT-care should have appropriate knowledge and procedures in place to ensure appropriate management of temporary OAT-cessation and re-initiation in the case of elective and invasive procedures - Audits of anticoagulation services should be undertaken using relevant quality indicators to assess the effectiveness of the service and impact on patient outcomes, and to identify opportunities for improvement - Audits should be performed by independent qualified auditors - Audits should be performed periodically, within predetermined periods of time - Anticoagulation services participating in the net of OAT-care should have their license renewed periodically based on audit results. Pharmacists, nurses, and physicians should be properly certified and regularly educated on OAT-management *(see also recommendation 18)* - Service users’ and caregivers’ feedback on service provision should be sought - Cost-effectiveness should be considered during framework development and review. Feedback procedures should not only consider the costs of OAT, but also the impact on the health care system (e.g., cost of monitoring and cost of complications that are prevented/ generated by means of therapy) - All aspects of the governance framework should be independent of the pharmaceutical industry | | | |
| **16. PHARMACEUTICAL CARE WORKFORCE** | **Mean LoA: 8.9±1.6** | **Median LoA: 9.0** | **Mean LoI: 1.3±0.4** |
| ***Main recommendation:***  **The pharmaceutical team (e.g., pharmacy) should have the right skill mix, capability, attitude and capacity to develop and provide safe, high-quality, and patient-centred services to OAT-patients, to prevent adverse outcomes (e.g., AF-related stroke, haemorrhage,deep venous thromboembolism, and pulmonary embolism).**  *Specifications:*   - The pharmaceutical team skill mix should meet the pharmaceutical care needs of patients and their caregivers, and ensure high-quality service, safety, and efficacy of OAT - Support and funding are essential in realising the optimal skill mix within a pharmaceutical team, capable of meeting the needs of both patients (irrespective of their cultural background) and providing effective anticoagulation services - Training records should be maintained and updated using regular competency assessments and revalidation processes - All pharmaceutical staff should have effective performance management, quality systems, and personal plan processes that encourage the delivery of safe and high-quality OAT-care - In each pharmaceutical team, staff members should be adequately trained in OAT-management to ensure appropriate service availability at all times *(see also recommendation 18)* | | | |
| **17. ROLE OF SPECIALIZED HEALTH CARE PROVIDERS** | **Mean LoA: 8.6±1.6** | **Median LoA: 9.0** | **Mean LoI: 1.4±0.6** |
| ***Main recommendation:***  **Specialized health care providers (e.g., pharmacists, physicians, and nurses) should offer ongoing support to OAT-patients.**  *Specifications:*   - Support should preferably be offered by specialized health care providers, trained in OAT-management and educational procedures, and as part of a multidisciplinary team - These health care providers should be regularly educated on OAT-management (e.g., guideline learning, lab test interpretation training, and training in working with blood samples) - The support of specialized health care providers to patients should be as standardized as possible and include: education and practical advice on OAT, measurement of INR values, monitoring of renal and liver function, monitoring and support of adequate medication use (adherence), education on potential consequences of non-adherence (e.g., haemorrhage, AF-related stroke, deep venous thromboembolism, and pulmonary embolism), advice on what to do in emergency situations, dose adjustments or suspensions (e.g., in case of surgery), and planning of next control visits - Specialized health care providers should specifically focus on the identification of high-risk situations, especially those caused by clinical changes and drug interactions - Pharmacists should also perform medication reconciliation and medication review at every transition in care *(see also recommendation 10)* - On a national level a network could be established to unite health care providers involved in OAT-care, and to stimulate knowledge exchange | | | |
| **18. CONTINUING PROFESSIONAL DEVELOPMENT** | **Mean LoA: 9.4±1.0** | **Median LoA: 10.0** | **Mean LoI: 1.3±0.5** |
| ***Main recommendation:***  **All team members involved in OAT-care should receive an appropriate level of Continuing Professional Development (CPD), including staff training, ongoing educational development, and documented competency assessment, to ensure safe and effective OAT-care.**  *Specifications:*   - CPD should be provided to all team members who prescribe, dispense, prepare, adjust dose, coach, monitor, and/or discharge OAT-patients, to ensure the necessary competency to undertake OAT-related duties safely - Well-structured CPD on OAT-care should be provided, based on needs assessment. Needs assessment could be performed through surveys, observations, and self-assessment - CPD should address the main areas of knowledge, skills (e.g., pharmacotherapeutic knowledge, interpretation of blood results, communication skills), and attitudes, to ensure adequate OAT-care - CPD should address the multidisciplinary character of OAT-care, and team members should be trained to work together with patients, their caregivers, and other health care providers to deliver safe and high-quality OAT-care - All health care providers should have an education plan indicating how often they will get their specific training (and examinations) on OAT-management - Staff training should be developed in a structural manner, with all team members to complete and/or pass their training prior to live OAT patient management - Besides patients and their caregivers, responsible health care providers should be educated on the term "adherence”, and how this impacts on OAT-patients | | | |

*LoA = Level of Agreement; LoI = Level of Importance*
